# HIV-1 CRF08_BC mutants resistant to reverse transcriptase inhibitors

**DOI:** 10.1186/1471-2334-14-S2-P60

**Published:** 2014-05-23

**Authors:** Hao Wu, Xiao-Min Zhang, Bo-Jian Zheng

**Affiliations:** 1 University of Hong Kong, Hong Kong, Hong Kong

## 

Human immunodeficiency virus type (HIV)-1 circulating recombinant form 08_BC (CRF08_BC), carrying recombinant reverse transcriptase (RT) gene from subtype B and C, has recently become highly prevalent in Southern China. As the number of patients infected by CRF08_BC increases, it is important to characterize the drug resistance mutations of CRF08_BC, especially against widely used antiretrovirals. In this study, clinically isolated virus was propagated in human peripheral blood mononuclear cells (PBMCs) with increasing concentrations of nevirapine (NVP), efavirenz (EFV) or lamivudine (3TC). Three different resistance patterns led by initial mutations of Y181C, E138G and Y188C were detected after in vitro selection with NVP. Virus variants with initial mutations, in combination with three other previously reported substitutions (K20R, D67N, V90I, K101R/E, V106I/A, V108I, F116L, E138R, A139V, V189I, G190A, D218E, E203K, H221Y, F227L, N348I and T369I) or novel mutations (V8I, S134N, C162Y, L228I, Y232H, E396G and D404N) developed during NVP selection. EFV-associated variations contained two initial mutations (L100I and Y188C) and three other mutations (V106L, F116Y and T139V). Phenotypic analyses showed that E138R, Y181C and G190A contributed high level resistance to NVP, while L100I and V106L significantly reduce virus susceptibility to EFV. Y188C resulted in a 20-fold reduction of susceptibility to both NVP and EFV. M184V was selected by 3TC as expected. This mutation, alone or with V90I or D67N, decreased 3TC susceptibility by over 1000 folds. These results have brought new insight into the development of drug-related mutations in patients and provided useful information for the optimization of antiretroviral regimens.

